# A general SNP-based molecular barcode for *Plasmodium falciparum *identification and tracking

**DOI:** 10.1186/1475-2875-7-223

**Published:** 2008-10-29

**Authors:** Rachel Daniels, Sarah K Volkman, Danny A Milner, Nira Mahesh, Daniel E Neafsey, Daniel J Park, David Rosen, Elaine Angelino, Pardis C Sabeti, Dyann F Wirth, Roger C Wiegand

**Affiliations:** 1 Broad Institute of MIT and Harvard, Cambridge, Massachusetts, USA; 2 Department of Immunology and Infectious Diseases, Harvard School of Public Health, Boston, Massachusetts, USA; 3 School for Health Studies, Simmons College, Boston, Massachusetts, USA; 4 The Brigham and Women's Hospital, Department of Pathology, Boston, Massachusetts, USA; 5 Systems Biology Program, Harvard University, Cambridge, Massachusetts, USA

## Abstract

**Background:**

Single nucleotide polymorphism (SNP) genotyping provides the means to develop a practical, rapid, inexpensive assay that will uniquely identify any *Plasmodium falciparum *parasite using a small amount of DNA. Such an assay could be used to distinguish recrudescence from re-infection in drug trials, to monitor the frequency and distribution of specific parasites in a patient population undergoing drug treatment or vaccine challenge, or for tracking samples and determining purity of isolates in the laboratory during culture adaptation and sub-cloning, as well as routine passage.

**Methods:**

A panel of twenty-four SNP markers has been identified that exhibit a high minor allele frequency (average MAF > 35%), for which robust TaqMan genotyping assays were constructed. All SNPs were identified through whole genome sequencing and MAF was estimated through Affymetrix array-based genotyping of a worldwide collection of parasites. These assays create a "molecular barcode" to uniquely identify a parasite genome.

**Results:**

Using 24 such markers no two parasites known to be of independent origin have yet been found to have the same allele signature. The TaqMan genotyping assays can be performed on a variety of samples including cultured parasites, frozen whole blood, or whole blood spotted onto filter paper with a success rate > 99%. Less than 5 ng of parasite DNA is needed to complete a panel of 24 markers. The ability of this SNP panel to detect and identify parasites was compared to the standard molecular methods, MSP-1 and MSP-2 typing.

**Conclusion:**

This work provides a facile field-deployable genotyping tool that can be used without special skills with standard lab equipment, and at reasonable cost that will unambiguously identify and track *P. falciparum *parasites both from patient samples and in the laboratory.

## Background

Nearly all malaria-associated mortality in children is due to infection with *Plasmodium falciparum*, which causes over 300 million clinical infections and a million deaths per year in African children under five years of age [1]. Genome sequencing of multiple parasite isolates [2-5] indicates that the parasite population is highly diverse. Genetic diversity in *P. falciparum *is manifested in the form of single nucleotide polymorphim (SNPs), microsatellite repeats, insertions, deletions and a range of gene duplication events. Much of this diversity segregates independently. Analysis of the progeny from a genetic cross suggests that the parasite genome is approximately 50-fold more 'recombinogenic' than the human genome [2]. This genetic diversity underlies the ability of the parasite to escape both immune clearance and drug treatment.

Parasite genetic variation can be exploited epidemiologically to provide a means of uniquely identifying parasites that infect individuals and to follow these parasites through the course of infection as drug or other interventions are applied. For example, these methods are useful for identifying parasites in an individual prior to treatment to later determine if post-treatment parasitaemia are the result of failure to clear the original parasites by the drug(s) (recrudescence) or of re-infection by another parasite form. These methods are critical for determining the efficacy of drugs in the field.

The first powerful and easily deployable tool for assessing the identity (i.e., genotype) of a given parasite isolated from a patient was published in 1994 by Snounou using three polymorphic loci (merozoite surface protein (*msp*)-1, *msp*-2, and glutamine rich protein, *glurp*) in a nested polymerase chain reaction (PCR) to identify length polymorphisms within six potential alleles (K1, MAD20, and RO33 for *msp-1*; IC and FC27 for *msp-2*; and *glurp*) [3]. The technique requires gel electrophoresis, fluorescent primers in conjunction with capillary electrophoresis to resolve the length of the individual allele polymorphisms, or a Luminex fluorescent microsphere assay [4]. The practical use of this genotyping system – whole or in part – was reviewed by Collins *et al *for 91 of 384 studies between 1995 and 2005, focused on antimalarial clinical trials [5]. They conclude from this meta-analysis that the great deal of variation found in the use and interpretation of this system was statistically related in multivariate analysis to the polyclonality of infections, treatment employed, geographic location, and duration of follow-up [5]. Pyrosequencing has also been used to evaluate short stretches of DNA sequence from patient samples surrounding a single locus to quantify alleles and haplotypes of MSP-1 in a population [6]. A modification of the standard MSP/GLURP genotyping technique, the heteroduplex tracking assay, demonstrated an ability to track complex infections in an endemic area with high polyclonality (3.82 per patient). However, this assay currently depends on the use of radioactive tracers, making it difficult or impossible to employ in many field settings [7]. Although others have offered refinements on these basic approaches or focused on microsatellite polymorphisms for determining polyclonality, or some combination thereof, the current approaches are either labour-intensive, difficult to carry out in a field setting, or require subjective interpretation [8,9].

As part of an ongoing effort to map genomic diversity of *P. falciparum*, genome sequencing has identified more than 112,000 SNPs from about 18 parasite genomes [10-12]. Current technology has made genotyping of SNPs by real-time PCR using dual probes in an end-point detection assay a standard practice. We filtered the discovered SNPs to create a panel of genotyping assays capable of defining a "molecular bar code" or signature for a given malaria parasite. Ideal SNPs for such an assay panel segregate independently, are common (i.e. exhibit a high minor allele frequency (MAF)) and are broadly distributed across the genome. The assay method has to be easy to use, inexpensive, and applicable to a wide variety of both field and laboratory derived material. The TaqMan system was choosen as a genotyping methodology, with the eventual goal of developing these assays into a simple, end-point PCR process that could be performed in the field where a PCR machine and a plate reader were available.

This work describes the first *P. falciparum *molecular bar code composed of 24 SNPs that in combination create a unique fingerprint or signature for a parasite genome. This methodology can be applied to a variety of laboratory and field samples including direct culture-adapted material, genomic DNA, frozen blood from patients or filter-paper collected samples with over 99% success. This methodology is extremely sensitive, requiring only a small amount of input material. Human DNA within the sample does not interfere with the results. This molecular barcode is also capable of identifying mixtures of parasite genomes within samples that would otherwise be identified as single parasite infections by conventional *msp-1 *and *msp-2 *genotyping, and thus provides a robust, inexpensive, facile method for evaluating parasite genomes within patient samples.

## Methods

### SNP selection and assay design

SNPs were chosen for assay development using two criteria – technical assessment of predicted assay performance and population MAF. All assayed SNPs were selected from a superset of approximately 2,100 SNPs previously discovered through comparative genome sequencing [10] which were found to be assayable on an Affymetrix genotyping chip (Neafsey *et al.*, manuscript in review). The initial set of SNPs was sent to Applied Biosystems (AB) for evaluation of the likelihood of TaqMan assay success. The SNPs determined to be most assayable technically were then examined for MAF in two populations: Senegal and Thailand. MAF was determined from a total of up to 22 patient isolates from Senegal and 16 patient isolates from Thailand. MAF was only calculated for SNPs that were successfully typed in at least five isolates from each population. SNPs exhibiting an average MAF of at least 35% across the two populations were chosen for final assay development so as to maximize coverage across chromosomes while maintaining a homogenous melting temperature (T_m_). SNPs were also assessed for their independence from one another to ensure that none of the loci were tightly linked. A total of 88 independent assays were designed and a final set of 24 assays were chosen to minimize the likelihood of obtaining identical bar codes from closely related isolates, while providing uniform quantitative performance. TaqMan Minor-Groove-Binder (TaqMan-MGB) probes were chosen for the MGB moiety's ability to offer improved performance with shorter probes through stabilization of the hybridized probe and subsequently higher reaction melting temperatures. The numbering corresponding to the 24 SNPs selected for this assay reflect the chromosome number and coordinate number of the 3D7 genome from version 5.0 of PlasmoDB[13].

### Sample preparation

To evaluate these methodologies under conditions as variable as those found in the field at malaria endemic sites, we used the following sample types: whole blood mixed with parasites in culture (either depleted or not depleted of white blood cells (WBCs) using PlasmodiPur), whole blood spotted on FTA paper from patients with smear-positive *P. falciparum *infection, frozen whole blood from infected patients (depleted or not of WBCs using PlasmodiPur), fresh and frozen parasite culture, residual wash from a vial of thawed parasites (previously frozen in glycerolyte), purified genomic DNA, and whole genome amplification (WGA) products both from purified genomic DNA and from patient samples described above. All samples were collected with informed consent under human subject guidelines and approved by the relevant institutional review boards. Historical culture adapted lines used in this study were evaluated by the institutional review board and deemed exempted under category 4 of the 45 CFR 46.101(b). For WGA samples, Qiagen REPLI-g (Catalog # 150045) was performed according to the manufacturer's protocol. Human DNA (Bioline Catalog # BIO-35025) was used as a control and mixed with various samples derived from *in vitro *culture to assess whether human DNA would interfere with assay results.

### DNA extraction

From FTA paper preserved whole blood samples, DNA was extracted using either QIAmp DNA Blood Mini Kit (Qiagen Catalog # 51106) or Gensolve blood spot kit (GenVault Catalog # GVR-50) using three-6 mm punches for each method. From whole blood samples that had been frozen, DNA was extracted using QIAmp DNA Blood Mini Kit (Qiagen Catalog #51106) and Promega Maxwell 16 Blood DNA Purification Kit (Promega Catalog # AS1010). From parasite culture, DNA was extracted using Qiagen g-100 (Catalog # 13343), DNAzol Direct (Molecular Research Center, Inc. Catalog # DN131) or by simply placing an aliquot of culture directly into the reaction without any extraction method (i.e., parasitized red blood cells directly from culture were diluted 1:100 and 0.5 – 1 μl per 5 μl reaction was used).

### DNA quantification

To distinguish between the amount of DNA derived from human and that from malaria sources in a whole blood sample, we developed a quantification method, both for human DNA, and for *P. falciparum *DNA using the TaqMan technology. To identify sequences for the malaria probe, we used data available from the 3D7 reference malaria genome [13], the database of SNPs from three sources [10-12], and expression data from *in vitro *and *ex vivo *experiments [15]. PF07_0076, a 519 bp gene encoding protein of unknown function on chromosome 7, was selected and sequence data from 3D7 was used to design TaqMan-MGB primers (forward: CGACCCTGATGTTGTTGTTGGA; and, reverse: GGCTTTTTTCCATTTCTGTAGTTAAGATTCA) and reporter sequence (CAACAGCTCCAAAATAT) probes from the highly conserved region of the gene.

HB3 genomic DNA was used as control in a series of 8 different dilutions: 30 ng, 10 ng, 3 ng, 1 ng, 0.3 ng, 0.1 ng, 0.03 ng, and 0.01 ng of DNA (based on OD_260_) per 5 μl reaction. All quantification was done in triplicate. Samples were processed for quantification depending upon the type of specimen. Purified genomic DNA from culture was quantified by either the picogreen or spectrophotometric assay and diluted to approximately 0.4 ng/μl in sterile water for optimal amplification signal; 2.5 μl was used per reaction. Purified DNA from blood spots was diluted 1:10 and 1 μl per 5 μl reaction was used. Parasitized red blood cells directly from culture or from fresh or frozen blood were diluted 1:100 and 0.5–1 μl per 5 μl reaction was used.

A master mixture was prepared using 2.25 μl Master Mix (Applied Biosystems Catalog # 4364343) and 0.250 μl of 20× Pf07_0076 pre-mixed quantification assay was used per reaction plus 10% to account for potential pipetting loss. All reagents were stored on ice with a foil cover while preparing mixes and dilutions. Controls and samples were loaded into 384-well PCR plates (total volume of DNA and water was 2.5 μl in a 5 μl reaction) followed by addition of the master mixture. Plates were loaded into an AB 7900 HT and run for Absolute Quantification: 50°C for 2 minutes, 95°C for 10 minutes, 95°C for 15 seconds, 60°C for 1 minute, repeat steps 2–4 for 40 cycles. For low DNA concentrations (e.g., blood spots or direct culture), cycle number was increased to 50.

Quantification of human genetic material was accomplished using Applied Biosystem's commercially available RNase P Control Reagent (AB Catalog # 4316844). A standard curve at the same concentrations previously discussed was generated using human genomic DNA from Bioline (Catalog # BIO-25025).

### *msp-1 *and *msp-2 *genotyping

*msp-1 and msp-2 *genotyping was performed in triplicate using the standard nested PCR method [3]. Briefly, 1 μl of sample (or about 5 ng of gDNA) was amplified with primers for the *msp-1 and msp-2 *loci with products approximately 900 and 700 bp. In the second round of PCR, internal primers for the three alleles of *msp-1 *(i.e. K1, MAD20, and RO33) and the two alleles of *msp-2 *(i.e. FC27 and 3D7/IC) were performed in separate reactions. The resulting products were resolved by electrophoreseis through 1.5% agarose gel in 1× TBE and scored for the number of alleles per sample.

### Bar-coding assay

Following quantification, samples were normalized for concentration with the minimum parasite DNA concentration per well for successful calling of all 24 SNPs in the molecular bar code as low as 1 pg per 5 μl reaction. For each reaction, template and water in a total volume of 2.5 μl was added to a 2.5 μl mix made up of 0.125 μl 40× SNP assay and 2.5 μl Master Mix (AB Catalog # 4364343) in a 384-well optical PCR plate and mixed, for a total reaction volume of 5 μl. The plate was covered with an optical plate seal and amplified in an ABI 7900 HT (ABI standard PCR protocol: 95°C for 10 minutes, 95°C for 15 seconds, 60°C for 1 minute, repeating steps 2–4 for 40 cycles). For samples containing low DNA concentrations (e.g. blood spots or direct culture), cycles were increased in number to 50. Following amplification, the samples were analyzed using Applied Biosystem's proprietary Allelic Discrimination and Absolute Quantitation software included in their SDS 2.x software suite using both 2.2.2 and 2.3.x versions. A good assay result for allelic discrimination trials was determined when the scattergram showed clearly separated clusters distinct from negative controls included on every plate. Successful results for quantitation were determined when the amplification curve had the anticipated log-growth curve for one or both alleles (in mixtures) that was clearly distinct from the background controls. A complete protocol can be found as Additional File 1.

## Results

**Figure 1:**
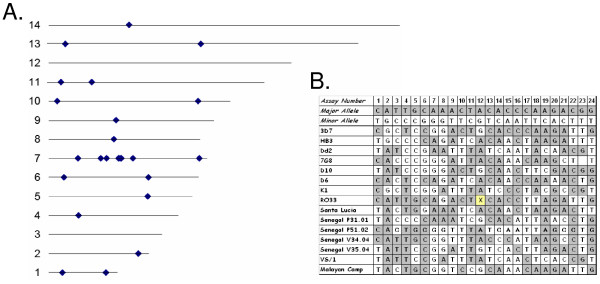
**Distribution of single nucleotide polymorphisms assayed and resulting molecular bar codes for sequenced parasites**. Twenty-four SNPs with an average minor allele frequency of at least 35% that were unlinked and assayable by TaqMan technology were selected from SNPs identified across the *P. falciparum *genome from sequencing efforts. The positions of the SNPs on the 14 chromosomes of *P. falciparum *are shown in A, with the sequence of the major and minor allele for each of the SNPs that comprise the molecular bar code shown in B. The position numbers in B (1 to 24) correspond to the positions in A, beginning with chromosome 1 through chromosome 14 and on each chromosome starting with the lowest coordinate number [13] and proceeding to the highest coordinate number on that chromosome (See Additional File 10). The major allele is indicated by a gray box and the minor allele by a white box. The yellow box (with an X) indicates no amplification. In all cases the TaqMan assay results matched sequence information.

Leveraging the large amount of sequence data derived from numerous parasite genomes, we identified a set of SNPs which were common among the sequenced parasites. Briefly, a set of unlinked SNPs from broadly distributed genomic locations was selected with an overall average minor allele frequency that exceeded 35% (Figure 1A and Additional Files 2 to 4). The "molecular bar code" is a collection of 24 of these markers that can be assayed simply and inexpensively using TaqMan technology (Figure 1B and Additional Files 2 to 4). For the development and evaluation of these assays, a method to quantify both parasite and human DNA within mixtures was required. Thus, a quantification probe for *P. falciparum *based upon the PF07_0076 gene was developed and a commercially available human quantification probe based on RNase P was used.

The parasite quantification probe corresponds to a region of the PF07_0076 gene (see Materials and Methods), which exhibits low genetic variation and is a single copy gene within a variety of parasites derived both from laboratory culture and patient samples [15]. For the purposes of standardizing the quantification, a parasite DNA standard using HB3 DNA that was used as a control for all assays in the analysis was created. Quantitation of this reference DNA sample was linear over a large quantitation range and was generally comparable to both standard pico green and spectrophotometric methods. This method has the advantage of being able to quantify *P. falciparum *DNA within a patient sample that contains human DNA. Thus, the qPCR method showed more consistency for assessing *P. falciparum *DNA contributions from sample to sample and across dilutions of samples than the fluorescent or spectrophotometric methods. To confirm that the parasite and human quantification probes worked independently, we created standard mixtures of genomic DNA from *P. falciparum *and human and demonstrated that quantification within the mixtures was consistent with quantification of both pure parasite and pure human DNA samples. Thus, the TaqMan quantification probe for *P. falciparum *was reliable and consistent both in samples containing parasite DNA alone and in samples containing a mixture of parasite and human DNA (Additional File 5).

**Figure 2:**
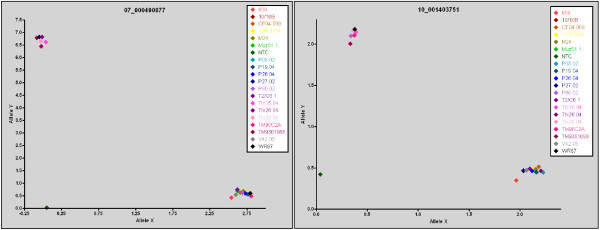
**TaqMan assays discriminate between the major and minor allele**. Two representative assays (corresponding to 07_000490877 and 10_001403751, with the remaining assays found in Additional Files 5 and 6) run for a subset of the parasites (corresponding to a typical running of the assay) are shown, indicating the clear separation between the signal derived from the major and minor allele. The major allele (Allele X) is displayed on the X axis and the minor allele (Allele Y) on the Y axis for 20 independent strains along with a non template control (NTC) containing only water.

**Figure 3:**
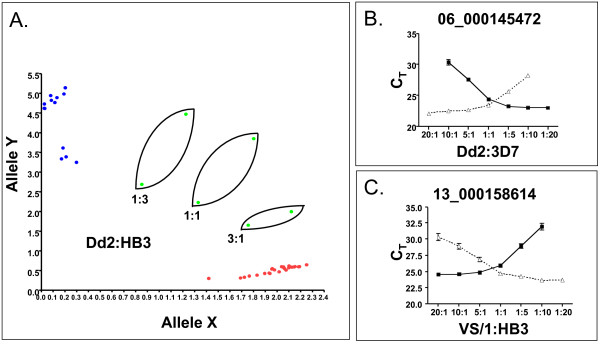
**TaqMan assays quantify alleles within DNA mixtures**. Relative amounts of each allele within a mixture of known amounts of indicated DNA were determined to correspond to the quantity of input DNA in the mixture. The relative ratios of Dd2:HB3 shown as green dots in panel A; and ratios for Dd2:3D7 and VS/1:HB3 shown on the X-axis for panels B and C, respectively. The major (red dots) and minor allele (blue dots) calls for 32 pure parasite DNA samples which type with either one or the other allele are indicated in Panel A. Panels B and C show the CT (threshold cycle) for the relative ratios of the indicated DNA samples for the 06_000145472 and 13_000158614 assays respectively. The closed squares indicate amplification of the allele labeled with VIC^® ^dye and the open triangles indicate amplification of the allele labeled with FAM™.

DNA samples from 12 sequenced parasites were used to validate the original 88 candidate bar code assays (Figure 1B). Assays were discarded for poor or late amplification or lack of ability to detect either allele. From this reduced set, individual assay performance to discriminate the major and minor allele was assessed (Figure 2 and Additional File 6,7), and each of the 24 assays showed clear differentiation of the two alleles for each position. The bar code provided robust and reliable discrimination of alleles in mixed genome samples (Figure 3). To evaluate specific performance in mixtures of known ratios, DNAs from two sequenced parasites – HB3 and Dd2 – were mixed in various proportions. Assays where the major and minor alleles were distinct were assessed for their ability to detect the minor allele in the mixture (Figure 3A). In mixtures of these DNAs ranging from a 3:1, 1:1 or 1:3 ratios, the assays were clearly able to quantify the proportion of the major and minor allele within the mixture. The ability of the assays to perform in mixed genome samples was evaluated by mixing DNA from sequenced parasites at known concentrations (1:20, 1:10, 1:5, 1:1, 1:5, 1:10, 1:20) to test the ability of the assay to detect both alleles (Figure 3B and 3C). Assays unable to detect both the major and minor allele, or an individual allele representing at least 20% of the total alleles in the population were also removed from consideration (Additional File 8). The final criterion for inclusion was that the SNPs assayed in the final bar code were broadly distributed across the genome and not in linkage disequilibrium with each other. To ensure that these assays were unlinked and independent, we performed linkage disequilibrium analysis (Additional File 9), and only unlinked loci were included. The final set of 24 SNPs selected for the molecular bar code meet all of the above criteria.

The molecular bar code assays were applied to 114 independent parasite lines (see Additional File 10) to identify samples with mixed genomes and derive unique signatures for the cultured parasite lines comprised of a single genomic background. The methodology worked on > 99% of samples requiring as little as 5 ng of genomic DNA. The utility of the bar code assay for patient-derived samples was determined by assessing its performance across a variety of sample types. DNA derived from culture-derived samples, from patient-derived samples, and from experimental samples were tested in the assay. For the culture-derived samples either genomic DNA derived from the parasite or direct culture material was used. For the patient-derived samples either blood spots dried onto filter paper or frozen blood samples were used. The experimental samples were mixtures of genomic DNA (parasite and human or multiple parasites), mixtures of culture with whole human blood, or WGA material from a variety of samples. The molecular bar code was robust and reliable for all of these sample types. In general a reliable bar code genotype was detected with less than 5 ng of genomic DNA from culture-adapted or patient-derived material. WGA products from a variety of samples (extracted DNA from culture or patient samples) also reliably and reproducibly provided a molecular bar code with the minimum input of ~1 ng DNA.

**Figure 4:**
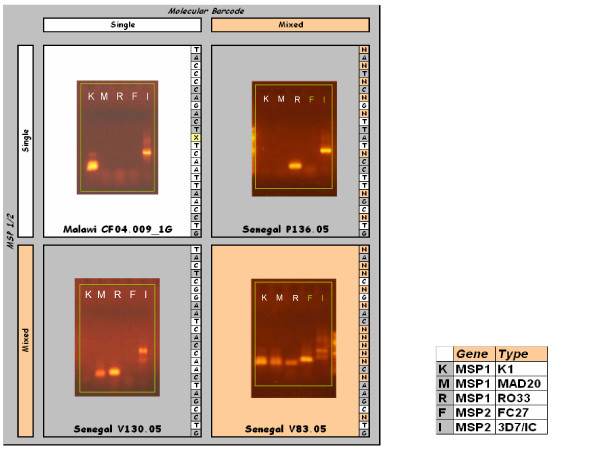
**Comparison of MSP-1 and MSP-2 genotype with molecular bar code assay**. Patient samples from Senegal and Malawi were genotyped using standard MSP1/2 methods. Examples of samples where both molecular bar code and MSP1/2 methods matched are shown in the upper right (single genome, white background) and the lower left (mixed genomes, orange background), with unmatched examples shown in the upper right and lower left (gray background). Samples having a single product for each of MSP-1 and MSP-2 were shown to have a single bar code (top left, white background) and a mixed bar code (top right, gray background). Mixed MSP-1 and MSP-2 samples were shown to have a single bar code (bottom left, gray background) and a mixed bar code (bottom right, orange background) using the molecular bar code technology.

The molecular bar code's utility for identifying mixed parasite samples was evaluated relative to standard MSP-1 and MSP-2 genotyping (Figure 4). MSP-1 and MSP-2 represent highly polymorphic surface molecules within the parasite that vary in length, and thus can provide a measure of whether the sample has a mixture of parasite genomes. In most cases the two methods were in agreement, but a few cases were found where MSP-1 and MSP-2 genotyping indicated that the sample contained a single genome while the bar code demonstrated a mixed genotype or conversely, a mixed genome sample by MSP-1 and MSP-2 had a single genotype by the molecular bar code method. A collection of 61 patient samples were tested and compared for their findings of "single" or "mixed" genome using MSP typing or molecular bar code methods. Of these 61 samples, 35 (57.3%) were single/single (MSP/molecular bar code); 5 were single/mixed (8.2%); 5 were mixed/single (8.2%) and 16 were mixed/mixed (26.5%) [Pearson's chi-squared value of 24.74, with a p-value of < 0.001]. In using the bar code assay to track parasites in patient samples as they are being adapted to culture, it was observed that individual parasite genomes as indicated by single alleles at each of the 24 assays in the molecular bar code could be isolated from these mixtures during subcloning. It was also observed that MSP-1 and MSP-2 genotyping can occasionally detect a second genotype with greater quantitative sensitivity than the bar code assay, presumably due to fact that MSP-1 and MSP-2 genotyping involves a nested PCR methodology. The molecular bar code provides greater power to discriminate among strains than MSP-1 and MSP-2 genotyping because many more possible alleles exist among the 24 markers than length polymorphisms within these regions, and provides a clear, objective and reproducible result.

## Discussion

For both laboratory and field-based studies, it is of paramount importance to be able to identify and track parasites. In the field, a simple and inexpensive method to identify parasites within a patient sample pre- and post-treatment is critical for epidemiologic studies and therapeutic efficacy trials. In the lab, problems of mis-identification and cross-contamination have plagued both mammalian tissue culture [16, 17] and parasite tissue culture for decades [18, 19]. For example, it has been previously noted that the 106/1, FCB and FCR3 *P. falciparum *lines are nearly identical genetically, quite inconsistent with their presumed geographic origins. Establishing the provenance of cultured parasites and maintaining their integrity is essential to studies that depend on proper geographic identity and/or clinical observations attributed to those parasites.

This work describes the development of a molecular barcode assay that leverages genome-wide diversity data to create a concise and unique signature for every parasite genome. This field-deployable method employs 24 TaqMan genotyping assays of independently segregating SNPs sampled from across the genome, and requires only a small amount of easily collected material such as filter-paper-collected blood spots. The same SNPs can, of course, be assayed by any other available method. The TaqMan system was chosen for its robustness, potential to deploy with common laboratory equipment, and straightforward data interpretation.

Although this method is based upon currently available sequence data, it is inherently adaptable in that new genomic diversity information from yet-unsampled *P. falciparum *populations can be used to generate assays suited for strain identification in those populations in the event the current method is insufficient. Although the assays were designed based on data from Africa and Thailand, the chosen set of SNPs also performed well to distinguish parasites from Brazil. This method can also flexibly incorporate assays for phenotypes of interest, such as SNPs in *pfcrt *and *dhfr *known to directly confer drug resistance, or assays at non-polymorphic residues that quantitatively assess copy number of genes such as *pfmdr1 *that have been associated with some forms of drug resistance. As new markers of different phenotypes are identified through QTL or association studies, this simple method can be extended to include these new markers. Given that this method is simple, inexpensive and field-deployable, it can greatly enhance our ability to follow epidemiological or therapeutic efficacy trials. Furthermore, this simple and inexpensive assay system can be used in the laboratory to follow parasites through the culture-adaptation and subsequent subcloning processes to ask questions about selection and competition as parasites grow in mixtures.

As the molecular bar code is applied and refined it will be critical to continue to make comparisons with standard genotyping currently in the field including the MSP-1, MSP-2, GLURP combination or microsatellite genotyping. Though these initial assays are able to distinguish the relative proportions of major and minor alleles in simple mixtures, we need to improve upon the ability to detect multiple genomes within patient samples. Among culture-adapted parasite samples the molecular bar code was useful for tracking parasites during the culture-adaptation and subcloning process. Finally, this method was easily able to detect instances of cross contamination in the laboratory, thus providing a powerful quality control step within the laboratory setting.

## Conclusion

In conclusion, TaqMan technology is a methodology that allows rapid, reliable, and inexpensive genotyping of alleles across the *P. falciparum *genome, and may be applied to genotyping genetic loci that are important for drug resistance, invasion, virulence, and immunity. This methodology has been applied to develop a molecular bar code that provides a unique signature for the purposes of tracking parasites in the laboratory and within patient samples. This method provides a uniform and reliable method to genotype the identity of parasites when compared to conventional MSP-1 and MSP-2 genotyping, is able to identify mixtures of alleles in a sample that would otherwise be classified as containing a single parasite genome. This methodology can be applied to the identification of drug resistance mutations and for genotyping parasites in drug treatment trials to evaluate for recrudescence or reinfection.

## List of abbreviations

SNP: Single Nucleotide Polymorphism; DNA: Deoxyribonucleic Acid; MAF: Minor Allele Frequency; PCR: Polymerase Chain Reaction; AB: Applied Biosystems; T_m_: Melting Temperature; MGB: Minor Groove Binder; WBC: White Blood Cells; WGA: Whole Genome Amplification; EDTA: Ethylene Diamine Tetraacetic Acid; TBE: Tris Boric Acid EDTA; MSP: Merozoite Surface Protein; GLURP: Glutamine Rich Protein

## Authors' contributions

RD designed the quantification assay, designed and implemented the molecular bar code assay, and applied the technology; DAM designed the quantification assay, performed MSP-1 and MSP-2 genotyping and helped write the manuscript; NM helped carry out the MSP-1 and MSP-2 genotyping; SKV conceived of the experiment, guided assay development and worked with RD to troubleshoot the assays and wrote the manuscript; DEN and DP helped with minor allele frequency analysis to help select the alleles for assay design; DR prepared the DNA samples and helped implement the molecular bar code assay; EA and PCS carried out the linkage analysis for the assays; DFW and RCW conceived of the experiment, selected the SNPs for TaqMan assays and helped guide the assay development and application, and helped write the manuscript.

## Supplementary Material

Additional file 1 **Protocol for quantification and molecular bar code**. A detailed protocol of how to perform the quantification and molecular bar code assays described in this manuscript are provided. Click here for file

Additional file 2 **Overview of the molecular bar code 1**. Twenty-four SNPs (indicated by the black and white diamonds) were selected from over 112,000 SNPs discovered by sequencing (Volkman, 2007) and genotyping (Neafsey, submitted). SNPs were distributed across the genome, exhibited a high minor allele frequency (MAF), and were unlinked. TaqMan allelic discrimination assays were performed for the 24 SNPs to generate the "molecular bar code", which represents the sequences present at each of the 24 SNPs, listed in increasing order from chromosome 1 through 14. The assay 01_000539044 is shown as an example. The chromosomal position is circled in red and the split diamond symbol is shown to represent the A/G allele, for which the allele discrimination assay is shown, along with the resulting molecular bar code. The major allele at each position is shown in gray and the minor allele in white. Click here for file

Additional file 3 **Primer and probe sequences for 24 SNPs**. Sequences for the forward and reverse primers used to amplify the region surrounding the 24 SNPs that comprise the molecular bar code are shown, as well as the reporter probe sequences for the major or minor allele. The number of parasite genomes sequenced (Count) as well as the minor allele frequency (MAF) are indicated. Click here for file

Additional file 4 **Position of SNPs in molecular bar code assay**. The position of the SNPs detected by the 24 TaqMan assays in the molecular bar code are shown along with the chromosome (Ch) on which the SNP resides, and the position (coordinate number) on that chromosome, and the R/S number (if available). Information about the SNP type (Synonymous = S; NonSynonymous = NS; Intergenic = I); the degeneracy of the SNP (ND = non-degenerate; 2 = two-fold degenerate; 4 = four-fold degenerate); the coding Frame (1, 2, or 3); the Gene where the indicated SNP is located; the possible Alleles; the number of parasites sequenced (Parasites); and, the minor allele frequency (MAF) are also indicated. The allele; number of reads; and amino acid is also shown for the individual parasites sequenced. Click here for file

Additional file 5 **Quantification of parasite DNA**. Parasite quantification using the PF07_0076 parasite gene is not affected by human DNA within human to parasite mixtures from 1:1 down to 1:1000. The amount of HB3 input DNA in nanograms (ng) is indicated on the X-axis and the C_T _or threshold cycle is indicated on the Y-axis. The quantification done in the presence of either no human DNA (red diamonds), 1 ng of human DNA (yellow squares), or 10 ng of human DNA (green triangles) shows no difference when plotted. Click here for file

Additional file 6 **Allele discrimination data (individual) for all 24 probes on 20 isolates**. Allele discrimination assays run for a subset of the parasites (corresponding to a typical running of the assay) are shown, indicating the clear separation between the signal derived from the major and minor allele. The major allele (Allele X) is displayed on the X axis and the minor allele (Allele Y) on the Y axis for 20 independent strains along with a non template control (NTC) containing only water. Click here for file

Additional file 7 **Allele discrimination data (composite) for all 24 probes on 16 isolates**. Allele discrimination assays run for a subset of the parasites (corresponding to a typical running of the assay) are shown as a composite. The major allele (Allele X) is displayed on the X axis and the minor allele (Allele Y) on the Y axis for 16 independent strains along with a non template control (NTC) containing only water. Click here for file

Additional file 8 **Assay performance**. The performance of each assay was evaluated by assessing the lowest ratio (20:1 in dark green; 10:1 in light green; and 5:1 in red) where the X or Y allele could be detected in a mixture containing both the major and minor allele, with the reported performance for the assay being the highest ratio for detecting either the X or Y allele in a mixture. Click here for file

Additional file 9 **Alleles assessed in assay are unlinked**. Analysis of linkage disequilibrium among the bar code SNP markers for 52 parasites using data from a 3K Affymetrix Array (Neafsey, submitted). The plot shows a histogram of the pairwise correlation (R^2^) of 26 bar code SNP markers. The red line indicates the mean of the background distribution of R^2^, which is calculated from all pairs of SNP markers located on different chromosomes that were included on the Affymetrix 3k array, for the same group of samples shown in the corresponding histogram. The dotted and dash-dotted lines show one and two standard deviations away from the mean of the background distribution. The two outliers in a) are attributed to the SNP marker pairs Pf_07_000657939 and Pf_07_000671839 (R^2 ^= 0.4137) and Pf_000488164 and Pf_000490877 (R^2 ^= 0.5167). These SNP pairs are approximately 14 kb and 2.7 kb apart, respectively. As a result of this analysis, these two outlier assays were dropped from the final set of assays. Click here for file

Additional file 10 **Molecular bar codes and additional information for additional parasite samples evaluated**. Parasite samples, the geographic origin, the source, the molecular bar code, and the results of individual assays are indicated, with boxes shaded in gray representing the major allele; boxes in white representing the minor allele; boxes with an X shaded in yellow representing assay where no amplification was repeatedly observed; and, boxes with an N shaded in orange representing an assay where a mixed allele was observed. Click here for file
