# Supplementary material for: A general SNP-based molecular barcode for Plasmodium falciparum identification and tracking
Source: Malar J. 2008 Oct 29;7:223. doi: 10.1186/1475-2875-7-223 (PMC2584654; doi:10.1186/1475-2875-7-223)
Supplement: Additional file 1 — Protocol for quantification and molecular bar code. A detailed protocol of how to perform the quantification and molecular bar code assays described in this manuscript are provided. [file 1475-2875-7-223-S1.pdf]

For Quantification:

Required reagents:

- Applied Biosystems TaqMan-MGB 20x Assay designed for pf07_0076
- Applied Biosystems 2x MasterMix (TaqMan Universal PCR Master Mix, No AmpErase UNG, catalog number 4324018)
- Sterile water
- Sample templates for quantification
- PCR plates (96 well or 384-well)
- rt-PCR machine or thermocycler/plate reader combination
- Control template malaria DNA (concentration determined by OD_{260} or picogreen assay)

General Protocol for Quantification in 384-well plates (5ul total volume):

1. Make dilutions of control DNA:

We use 30ng, 10ng, 3ng, 1ng, 0.3ng, 0.1ng. 0.03ng, and 0.01ng of parasite DNA per reaction.

2. Make dilutions of sample DNA:

- For purified genomic DNA:
  - dilute stock tube 1:100 and use 1ul per reaction.

- For purified DNA from blood spots:
  - diute 1:10 and use 1ul per 5ul reaction.

- For parasitized red blood cells directly from culture or from fresh or frozen blood:
  - pellet red blood cells
  - dilute samples 1:100 and use 0.25-1ul per 5ul reaction.
  - the amount used per reaction depends on the color of the sample. Because samples with visible color will negatively effect the optics of the plate reader or rt-PCR machine, more dilution may be needed for darker samples.

- For DNA purified from FTA card blood spots:
  - use 5ul of sample if possible (do not dilute)

- For Whole Genome Amplified samples:
  - Dilute 1:10 and use 1ul per 5ul reaction.

3. Prepare Master Mix-assay mixture. Calculate the total volume of assay and Master Mix needed for the experiment, adding 15% more to the total volume to account for pipetting loss. Also remember to include at least one non-template (water) reaction. For 5ul reaction volumes using the 20x pre-mixed assay, use 2.25ul Master Mix and 0.250ul of assay per reaction. When preparing mixture, don’t allow assay to sit at room temperature or exposed to sunlight for

extended periods of time. If necessary, store on ice with a foil cover while preparing mixes and dilutions.

4. Load controls and samples into PCR plate. For a 5ul reaction, the total volume of DNA and water will be 2.5ul.

5. Load Master Mix-assay mixture into PCR plate containing templates. Mix well, taking care to avoid bubbles, especially at the bottom of the well. If necessary, after sealing plate, firmly tap plate on benchtop to dislodge bubbles and flash-spin plate (spin to 1,000rpm, then immediately stop centrifuge).

6. Load plates into thermocycler or rt-PCR machine.

Program for assay:

1. 50°C for 2 minutes (*optional:* We use MasterMix without UNG so omit this step)
2. 95°C for 10 minutes
3. 95°C for 15 seconds
4. 60°C for 1 minute
5. Repeat steps 2-4 for 40 cycles; increase to 50 cycles if expecting low DNA concentration (like blood spots or direct culture). PCR program should take approximately 1.5-1.75 hours. Remember to change reaction volume to 5ul.

<u>User Notes:</u>

All quantification is done in triplicate.

Assays are sensitive to light degradation as well as degradation due to multiple free-thaw cycles. Upon arrival, assays are thawed and aliquoted, then stored at -20°C. Stocks in use are stable at 4°C for up to a year if kept on ice and protected from light when in use.

If using ABI rt-PCR machine, select Absolute Quantification protocol.

Probe Information: pf07_0076, custom MGB assay from Applied Biosystems:
Concentration: 20x
Forward Primer Sequence: CGACCCTGATGTTGTTGTTGGA
Reverse Primer Sequence: GGCTTTTTTCCATTTCTGTAGTTAAGATTCA
Probe Sequence: CAACAGCTCCAAAATAT
Reporter: FAM
Quencer: NFQ

For Barcoding:

Required reagents:

• Applied Biosystems TaqMan Probe with MGB designed multiple SNPS, 40x, set of 24 assays

• Applied Biosystems 2x MasterMix (TaqMan Universal PCR Master Mix, No AmpErase UNG, catalog number 4324018)

• Sterile water

• Quantified templates for barcoding

• PCR plates (96 well or 384-well)

• rt-PCR machine or thermocycler/plate reader combination

General Protocol for Barcoding in 384-well plates (5ul total volume)::

1. Calculate DNA needed for reactions. We're currently using 24 assays, so make sufficient DNA dilutions for 30 reactions. Optimal DNA concentration is 1ng of quantified malaria DNA per reaction, but acceptable signals are returned for as little as 0.001ng of starting material. The volume of DNA and water per reaction is 2.5ul.

2. Dilute DNA in water.

3. Calculate volume of master mix and assay. When doing this calculation, add15% of the total reaction volume to account for pipetting loss. For each reaction, 0.125ul of pre-mixed 40x assay and 2.5ul of master mix are required. Make a stock tube for each assay to make pipetting more accurate. Given the small volumes of assay required, make stock for at least 10 reactions. When preparing mixture, don't allow assay to sit at room temperature or exposed to sunlight for extended periods of time. If necessary, store on ice with a foil cover while preparing mixes and dilutions.

4. Distribute template DNA into PCR plate (2.5ul each DNA template per well).

5. Distribute master mix-assay mixtures (2.5ul each mixture per well).

6. Mix well, taking care to avoid bubbles, especially at the bottom of the well. If necessary, after sealing plate, firmly tap plate on benchtop to dislodge bubbles and flash-spin plate (spin to 1,000rpm, then immediately stop centrifuge).

7. Load plates into thermocycler or rt-PCR machine.

Program for assay:

1. 50°C for 2 minutes (optional. We use the MasterMix without UNG so delete this step)
2. 95°C for 10 minutes
3. 95°C for 15 seconds
4. 60°C for 1 minute
5. Repeat steps 2-4 for 40 cycles; increase to 50 cycles if using low DNA concentration (blood spots or direct culture). PCR program should take approximately 1.5-1.75 hours. Remember to change reaction volume to 5ul.

If using ABI rt-PCR machine, make two protocols: one Absolute Quantification (for multicomponent data views) and the other Allelic Discrimination (for scattergram results).
