# Supplementary material for: A general SNP-based molecular barcode for Plasmodium falciparum identification and tracking
Source: Malar J. 2008 Oct 29;7:223. doi: 10.1186/1475-2875-7-223 (PMC2584654; doi:10.1186/1475-2875-7-223)
Supplement: Additional file 2 — Overview of the molecular bar code 1. Twenty-four SNPs (indicated by the black and white diamonds) were selected from over 112,000 SNPs discovered by sequencing (Volkman, 2007) and genotyping (Neafsey, submitted). SNPs were distributed across the genome, exhibited a high minor allele frequency (MAF), and were unlinked. TaqMan allelic discrimination assays were performed for the 24 SNPs to generate the "molecular bar code", which represents the sequences present at each of the 24 SNPs, listed in increasing order from chromosome 1 through 14. The assay 01_000539044 is shown as an example. The chromosomal position is circled in red and the split diamond symbol is shown to represent the A/G allele, for which the allele discrimination assay is shown, along with the resulting molecular bar code. The major allele at each position is shown in gray and the minor allele in white. [file 1475-2875-7-223-S2.pdf]

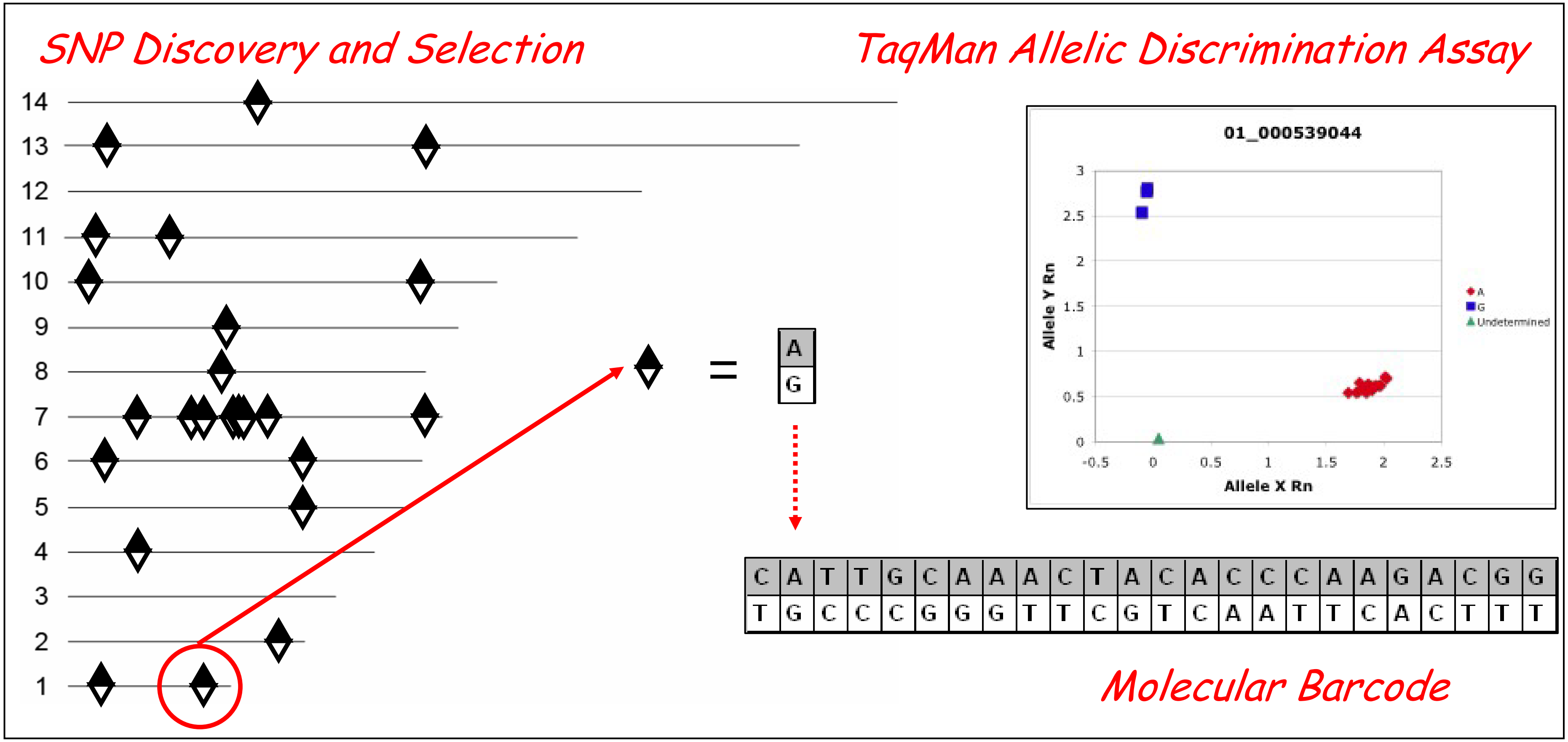

Daniels_Additional File 2
