# Supplementary material for: A general SNP-based molecular barcode for Plasmodium falciparum identification and tracking
Source: Malar J. 2008 Oct 29;7:223. doi: 10.1186/1475-2875-7-223 (PMC2584654; doi:10.1186/1475-2875-7-223)
Supplement: Additional file 3 — Primer and probe sequences for 24 SNPs. Sequences for the forward and reverse primers used to amplify the region surrounding the 24 SNPs that comprise the molecular bar code are shown, as well as the reporter probe sequences for the major or minor allele. The number of parasite genomes sequenced (Count) as well as the minor allele frequency (MAF) are indicated. [file 1475-2875-7-223-S3.pdf]

SNP	Count	MAF	Forward Primer Sequence	Reverse Primer Sequence	Reporter 1 Sequence	Repo
Pf_01_000130573	10	0.40	CTTCCTCTTACCCATATTTTCTTCTTTCCT	TGTGAAAATGAGAAGAGAGAAATATGTGTTGA	TCTTCATTATATGCATCTTTT	TTTCT
Pf_01_000539044	10	0.40	CCAAGAAGTCCAAGAATAATAGTTACTATCACA	TTCATGTGTGGCTGCTTTCCTAT	CAACCGTTTCTTTAGCC	CAACC
Pf_02_000842803	10	0.50	ATTCCAAAACTATGTTTGCTGCTTTTCA	AGAAATGCAGTGGTACTTGTTGCTA	AGATTATTTGCATCGTTACTT	AGATT
Pf_04_000282592	10	0.30	TGGTAATTTGTTTGATTGCTTACATTGTTATGT	GACAAGTTTTAATATGAACGGTCAGGTAGA	ACTCGTTCATGCTATTATA	ACTCG
Pf_05_000931601	10	0.30	CGATGACTACCATAATGATGTTCATATTCACA	TCCTCATTTCTGTTATTAAATTGTCATCATCTTCA	TTCGGGTGGTTGATCA	TCGGG
Pf_06_000145472	9	0.44	TGTTCATGTGTCCCCTCTTTTTCTG	GGAAGGTAGAATATCAAATACAAGGGATGT	AAGCATAGTAAGAAACATT	AAGCA
Pf_06_000937750	11	0.36	CTAGCTCAGCTTCCAATTTGTCAAA	GTCCACATCCATATACTGCTGTACAA	TGCATGTAGCAATACCCCCA	CATGT
Pf_07_000277104	11	0.36	AATTAATATTATGAATATGAGTACTCAGCATTATATCTT	ACGTCCATATGTTGAAGATGATTGCA	CCACCGAACTTATATTT	CACCG
Pf_07_000490877	12	0.42	CGTAAACCAGGAGTAAGACGTAAACT	AGATGAGTTAGCAACGAAACCATCAT	TTCCATTAGCTAAATTC	CATTA
Pf_07_000545046	10	0.30	GGATGTATGACAGCTTTTTCTAATTCTTCTCT	CCATCCTTTTATGAATGCTAAGAGAATACGT	TCTTAAGCCCGTTTAGTG	TCTTA
Pf_07_000657939	9	0.22	CGTCCACTTATGATAATGACCCTGAAA	TGCTTTCGTTTATATTGCAACATTTCTTCA	ACGATCGTCAAATTG	ACGAT
Pf_07_000671839	10	0.20	GGAACCATTCATAGTTTAGTAGAATCAACCA	AGTAGTCATGTGATATATGATTTTGTGGCAAA	CTTTTTCATTTTCATTTAATAAA	TTTTC
Pf_07_000683772	7	0.43	CTTTTATCGTACCACCATTAACATTTTGGA	ATAAAGATTTATCATTCGTTACAAATAACAATGAA	CATCATCATGATGGTTCAG	CATCA
Pf_07_000792356	9	0.22	TCTGTTTAATGGGACTCTTCTGTTTCG	ACTAAGTCCAATAGTTTGTATATTCCCTTGTTTT	ACCATACCAAATTTC	CCATA
Pf_07_001415182	16	0.50	AGGTACTGATTATTGGAATGATTTGAGCAA	ACTTTCCACCACTCATCACGAAAA	AGCTTATCATTTTTTTTATTCC	CTTAT
Pf_08_000613716	8	0.38	CACTTCAAATCTTTTCCAGTTATTTTCTATCCAT	TGGGAAAGAATTAACAATACTTATAAGTGCATAAATA	AAAAACTTCATTAAAAATCA	ACTTC
Pf_09_000634010	7	0.43	AATGTTAATTTTATTTCATCATCCGCATTTAATTT	AAATACCATGTGTACCTGTTGTGGAT	CCCTCAATCCACTTTTATA	CCCTC
Pf_10_000082376	4	0.50	TGTCGTTATTAAAAATATTTAAAGTTGACT	AGAAAGTATACATTTGTCCGCCGAA	ACGACATTGAAATTTTAT	ACGAC
Pf_10_001403751	9	0.33	ACAAGAACCCGTGGTACCTACATTA	CTGGTTTAGTGGATTCACCTTCAGA	AATGAAAACGTTACTCCC	ATGAA
Pf_11_000117114	7	0.43	ATTTTCATAAGAACTGCTTCCATCTCCTT	AAGTATCTTGCTCGTTTGTATAAAGCA	AAATTTCATCCAAAAGTAATT	TTTCA
Pf_11_000406215	12	0.42	ACGAATGAACAAAATTATGATACACAGGATGA	CTGATGACTTTTTTTCAAGTTCATTTTTGGATT	CTACAGCTAGTAATATTTC	CAGCT
Pf_13_000158614	7	0.29	AGTGTGAGCTTTCGTAAATATTTTAGTGAAGA	AGAGGATATCCAATAGGGTGCTGTT	ATTTTTGGACCCAAGCTA	ATTTT
Pf_13_001429265	12	0.42	CACACATATCAAAATCGCATTCATCCT	GAAAGGTATTCAAACCATAACAAAATGGATGA	CCTCCAGCAGTATATAC	CCTCC
Pf_14_000755729	10	0.40	CGTAACAAATCATCAATTAAGTCATCCGAAA	CAAATCTTGAGGAATAGGTTCATATGCTGAT	TTTTAAATTTTCAATTCTTTTTTG	AAATT
