# Supplementary figures and images for: A general SNP-based molecular barcode for Plasmodium falciparum identification and tracking
Source: Malar J. 2008 Oct 29;7:223. doi: 10.1186/1475-2875-7-223 (PMC2584654; doi:10.1186/1475-2875-7-223)

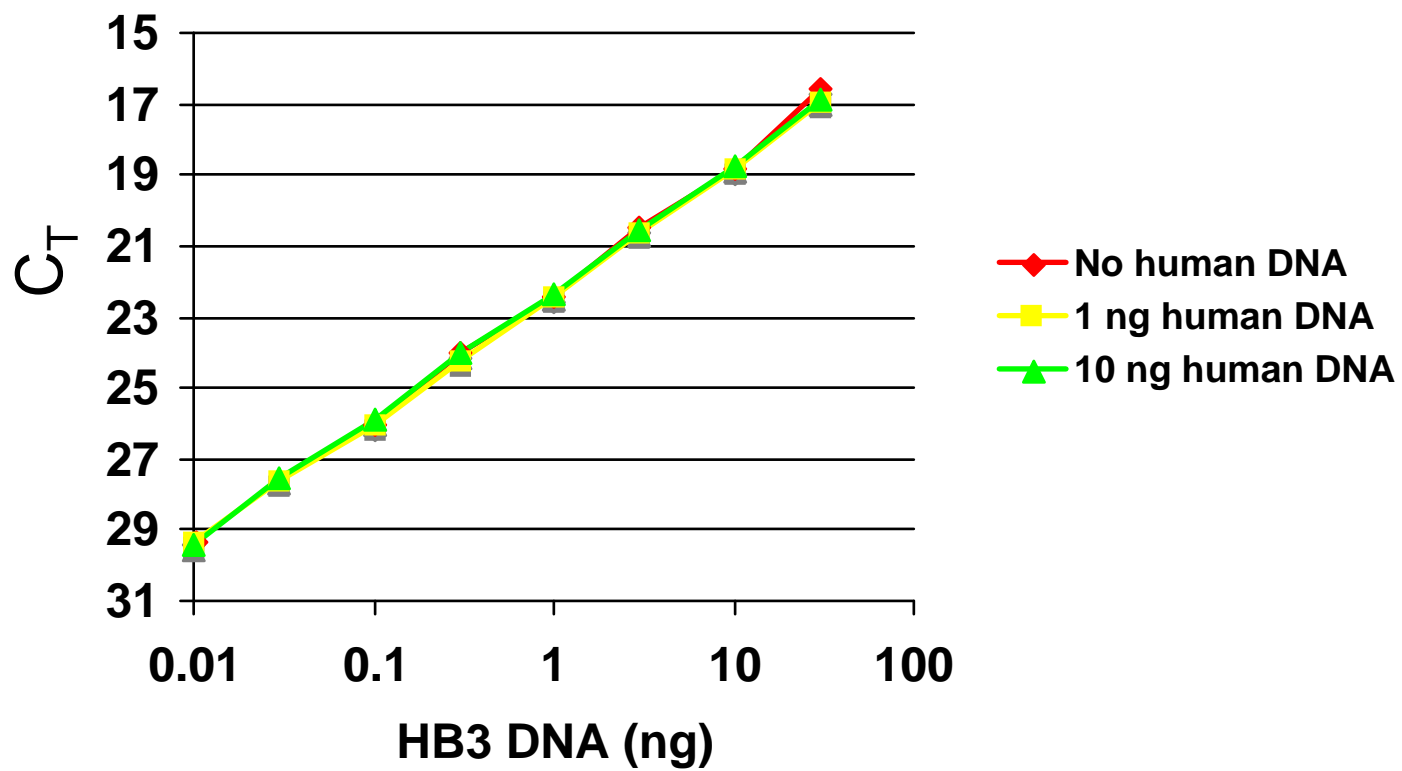

Daniels\_Additional File 5

Supplement: Additional file 5 — Quantification of parasite DNA. Parasite quantification using the PF07_0076 parasite gene is not affected by human DNA within human to parasite mixtures from 1:1 down to 1:1000. The amount of HB3 input DNA in nanograms (ng) is indicated on the X-axis and the CT or threshold cycle is indicated on the Y-axis. The quantification done in the presence of either no human DNA (red diamonds), 1 ng of human DNA (yellow squares), or 10 ng of human DNA (green triangles) shows no difference when plotted. [file 1475-2875-7-223-S5.pdf]

Relative Intensities for 16 Strains in 24 Assays

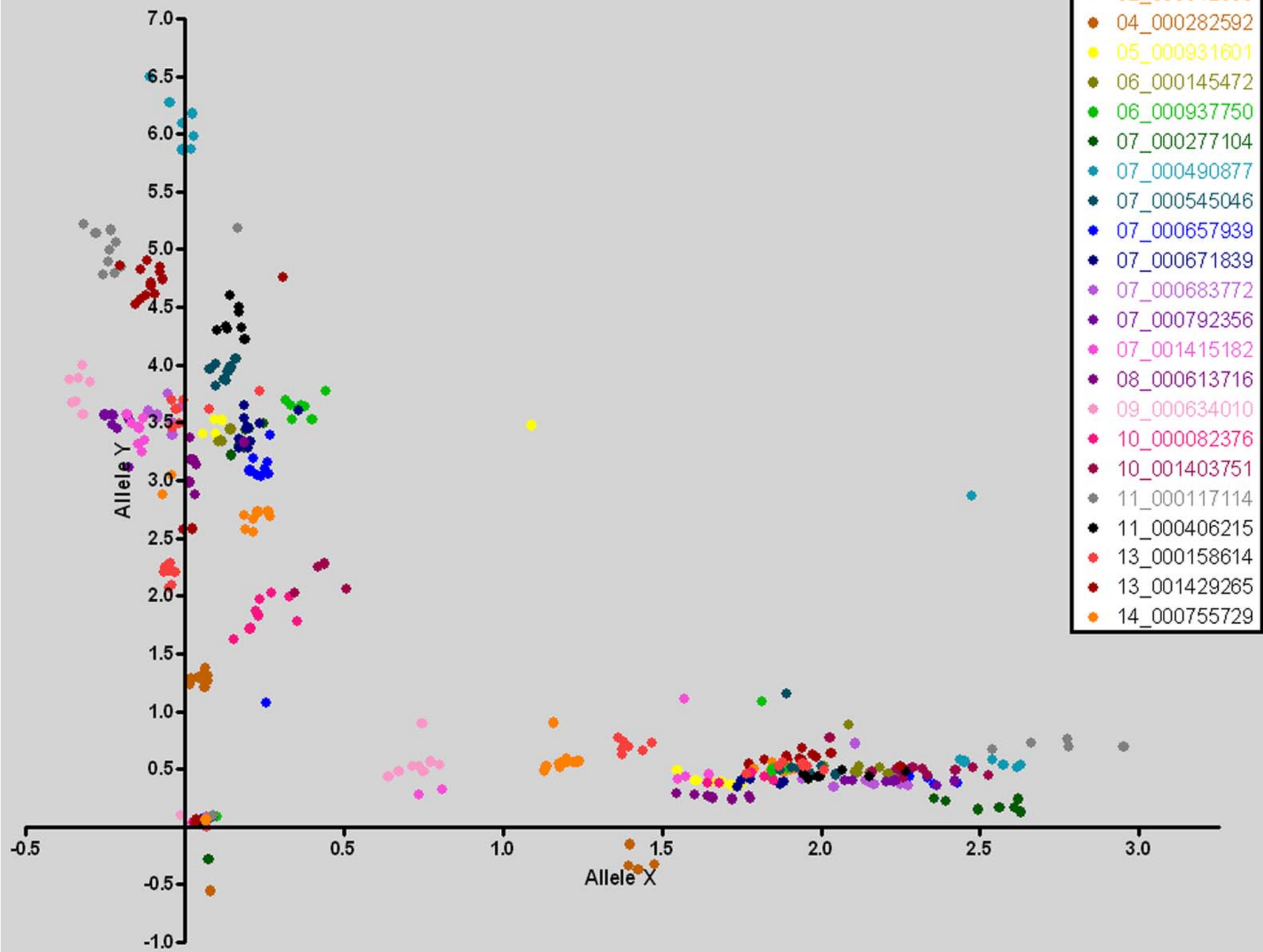

Supplement: Additional file 7 — Allele discrimination data (composite) for all 24 probes on 16 isolates. Allele discrimination assays run for a subset of the parasites (corresponding to a typical running of the assay) are shown as a composite. The major allele (Allele X) is displayed on the X axis and the minor allele (Allele Y) on the Y axis for 16 independent strains along with a non template control (NTC) containing only water. [file 1475-2875-7-223-S7.pdf]

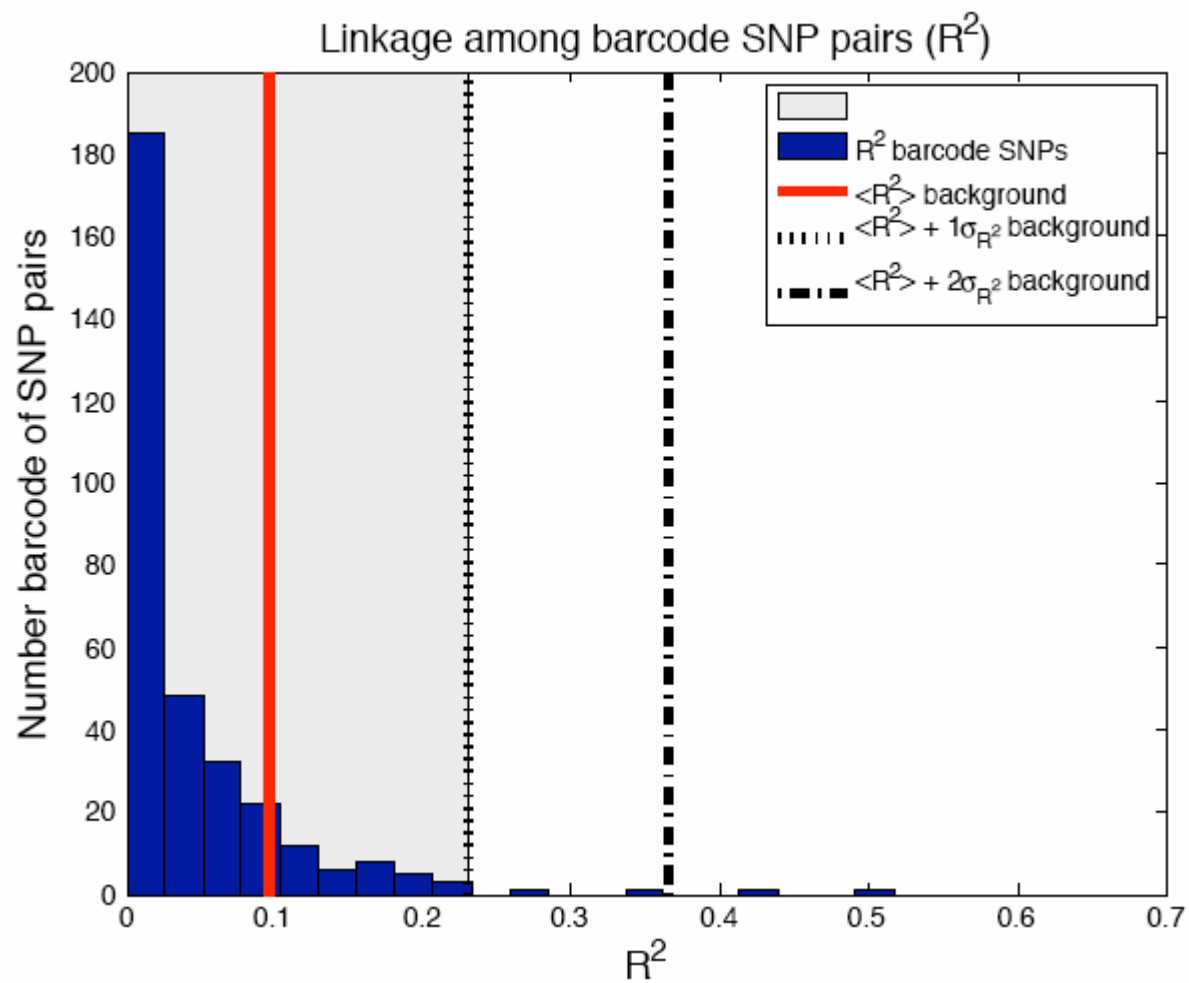

Daniels\_Additional File 9

Supplement: Additional file 9 — Alleles assessed in assay are unlinked. Analysis of linkage disequilibrium among the bar code SNP markers for 52 parasites using data from a 3K Affymetrix Array (Neafsey, submitted). The plot shows a histogram of the pairwise correlation (R2) of 26 bar code SNP markers. The red line indicates the mean of the background distribution of R2, which is calculated from all pairs of SNP markers located on different chromosomes that were included on the Affymetrix 3k array, for the same group of samples shown in the corresponding histogram. The dotted and dash-dotted lines show one and two standard deviations away from the mean of the background distribution. The two outliers in a) are attributed to the SNP marker pairs Pf_07_000657939 and Pf_07_000671839 (R2 = 0.4137) and Pf_000488164 and Pf_000490877 (R2 = 0.5167). These SNP pairs are approximately 14 kb and 2.7 kb apart, respectively. As a result of this analysis, these two outlier assays were dropped from the final set of assays. [file 1475-2875-7-223-S9.pdf]
