# Supplementary material for: A general SNP-based molecular barcode for Plasmodium falciparum identification and tracking
Source: Malar J. 2008 Oct 29;7:223. doi: 10.1186/1475-2875-7-223 (PMC2584654; doi:10.1186/1475-2875-7-223)

Relative Intensities and Allelic Discrimination: Assay 1 - 4

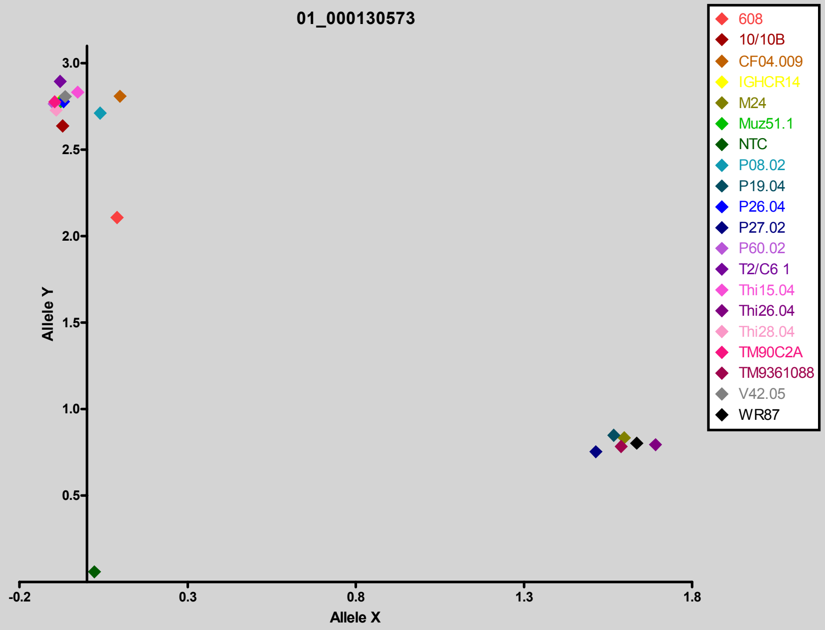

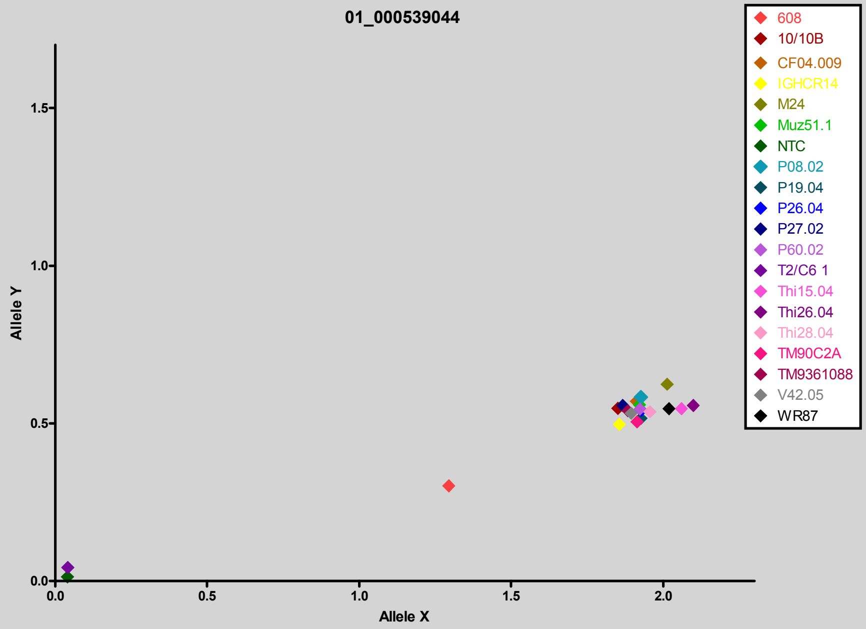

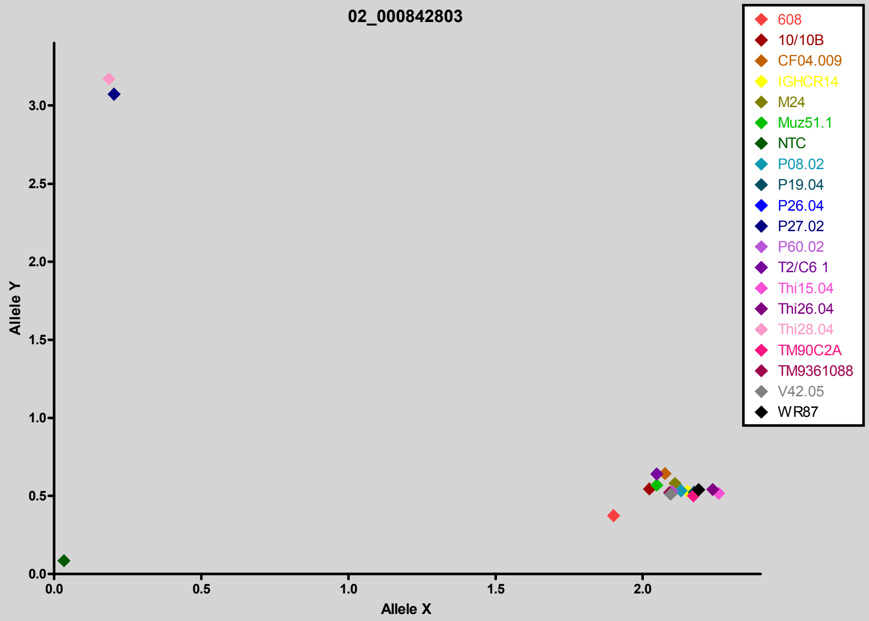

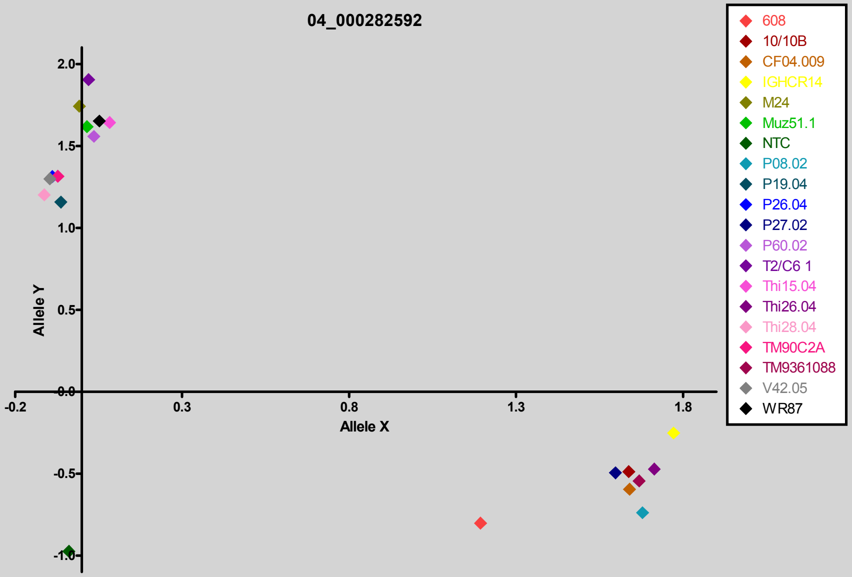

Relative Intensities and Allelic Discrimination: Assay 5-8

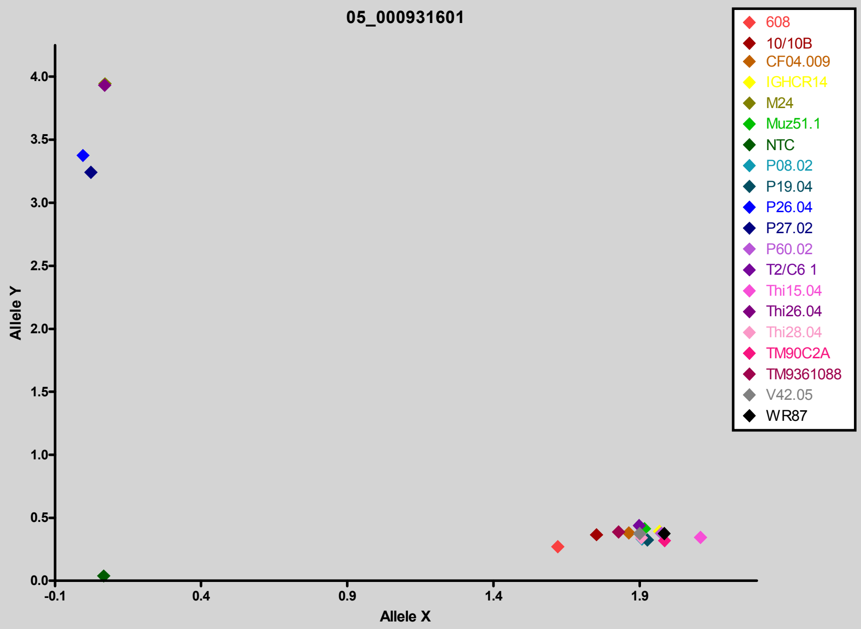

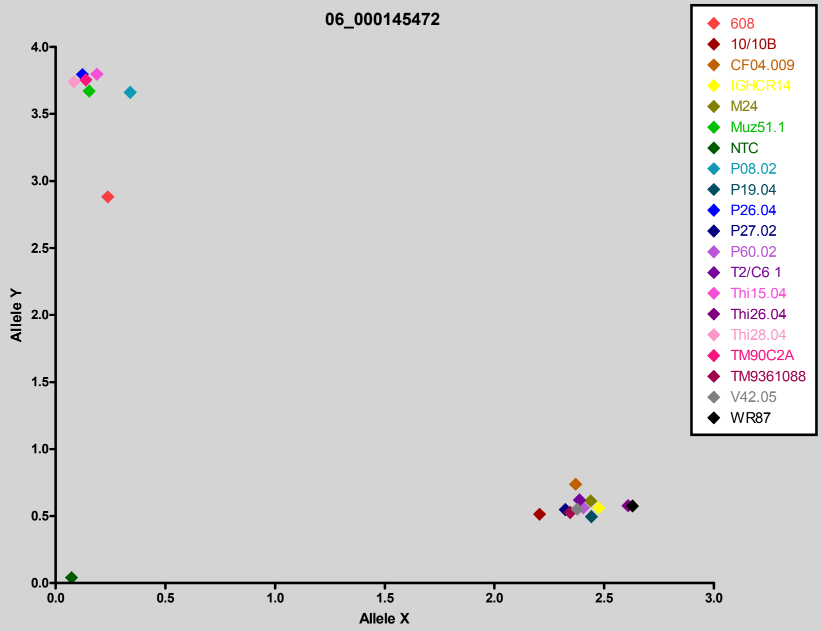

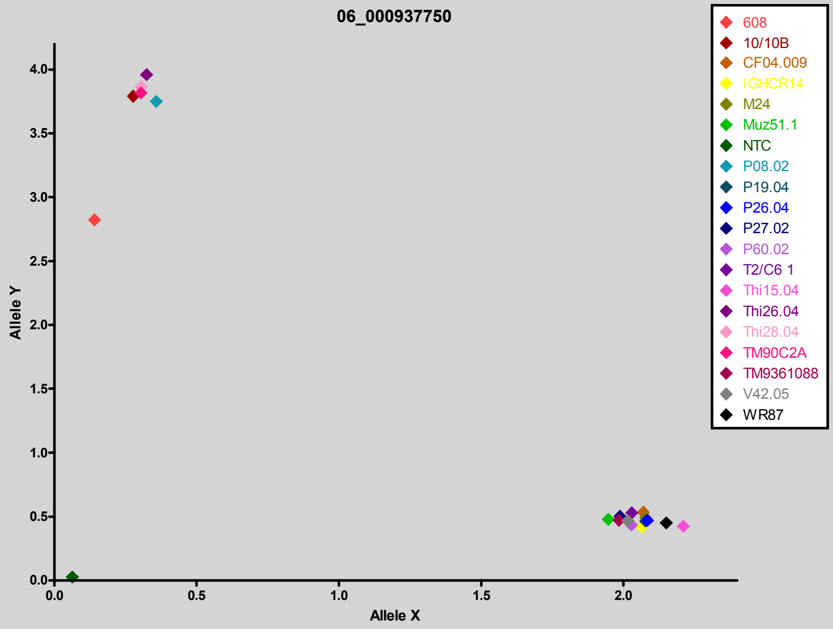

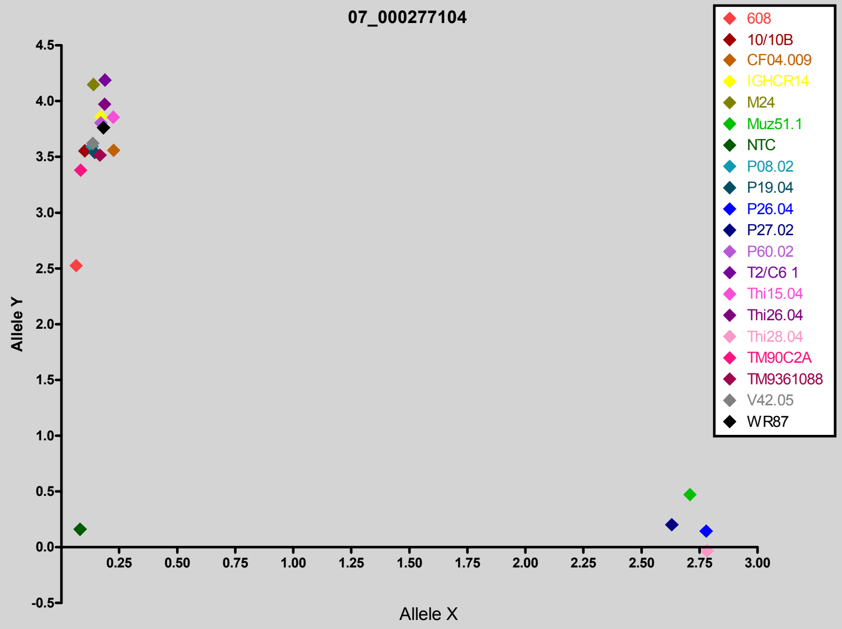

Relative Intensities and Allelic Discrimination: Assay 9 - 12

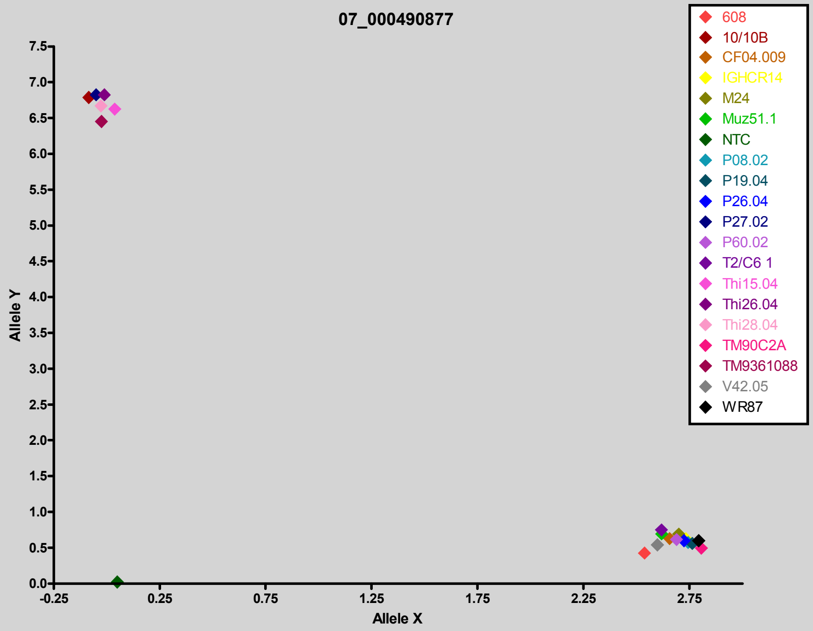

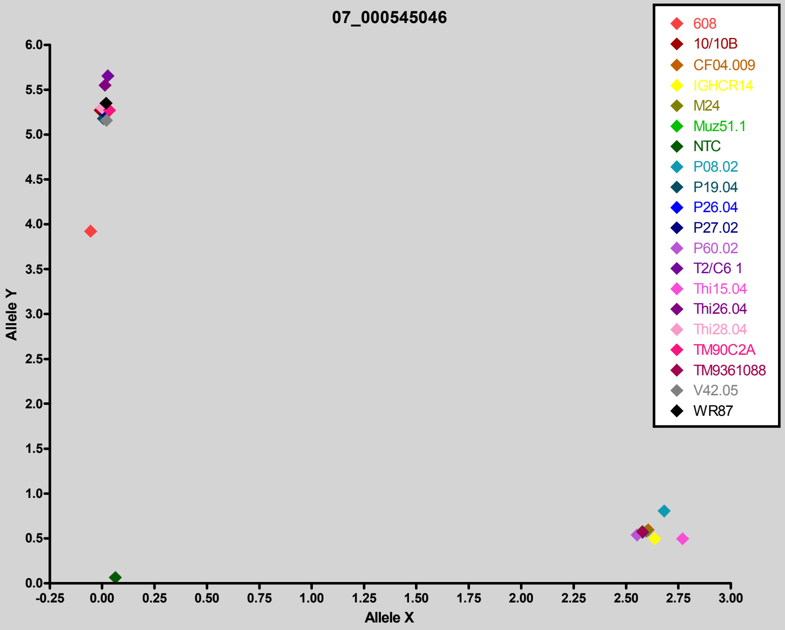

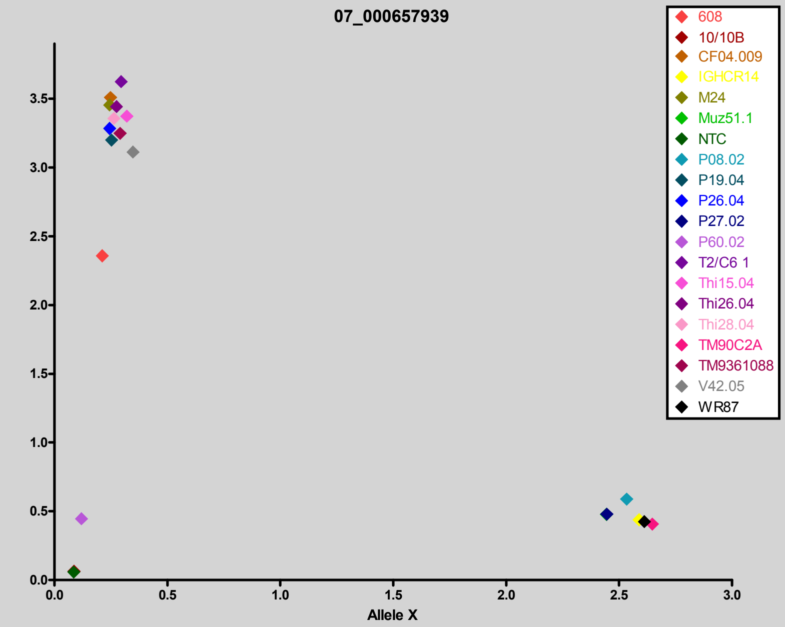

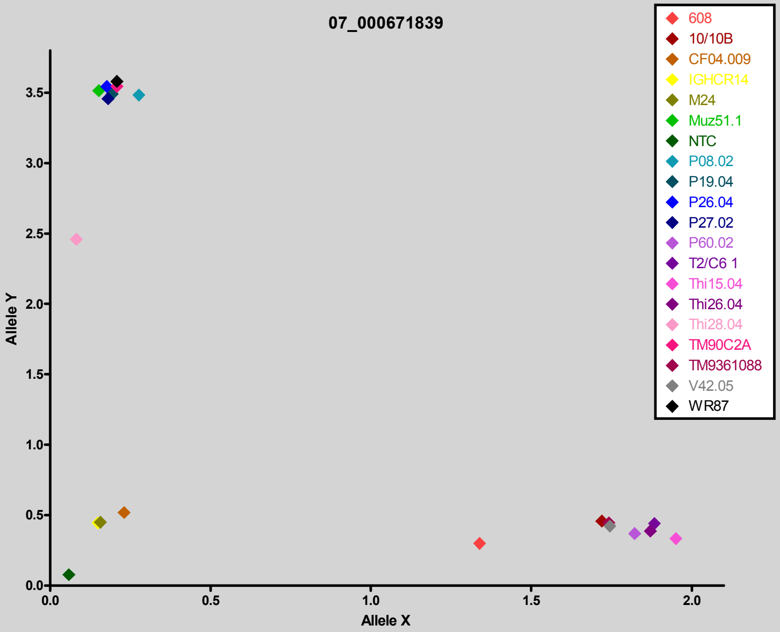

Relative Intensities and Allelic Discrimination: Assay 13 - 16

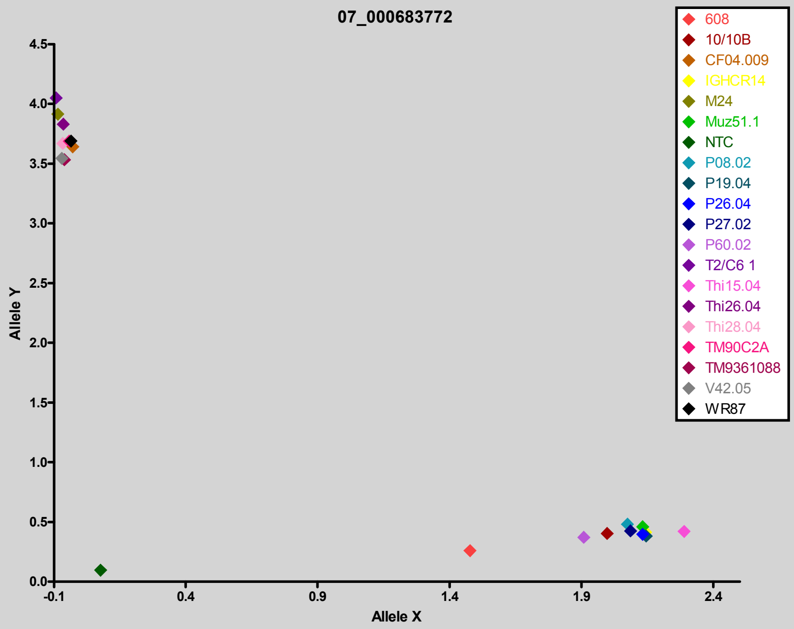

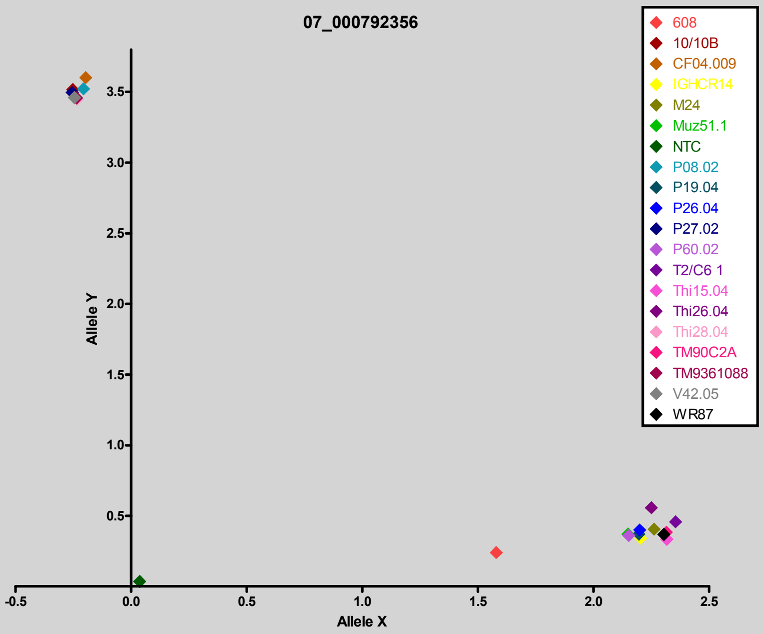

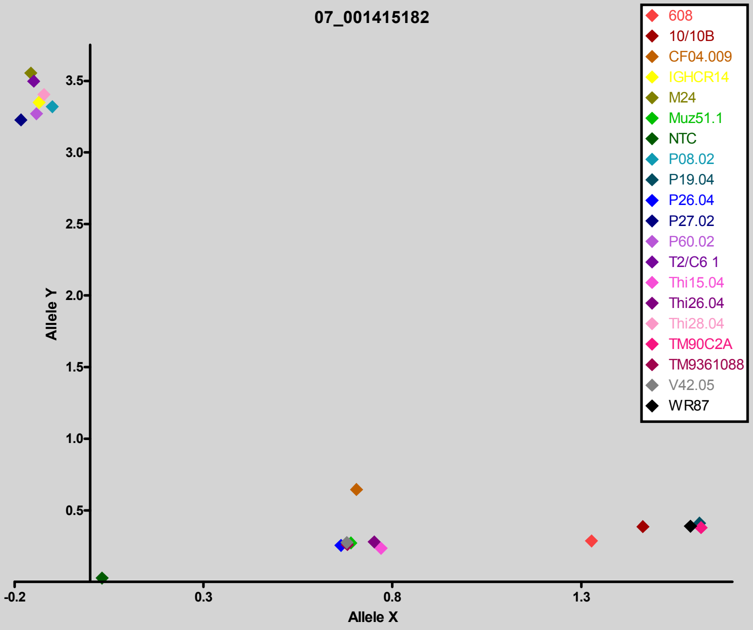

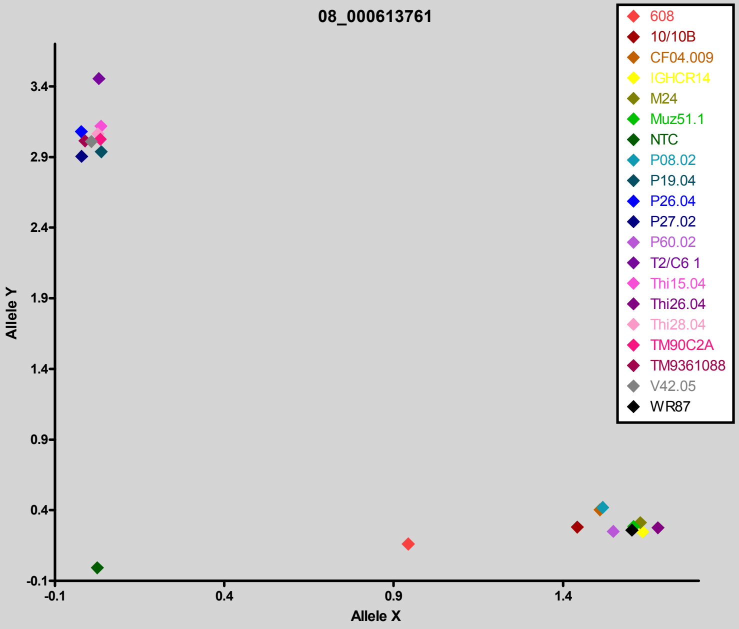

Relative Intensities and Allelic Discrimination: Assay 17 - 20

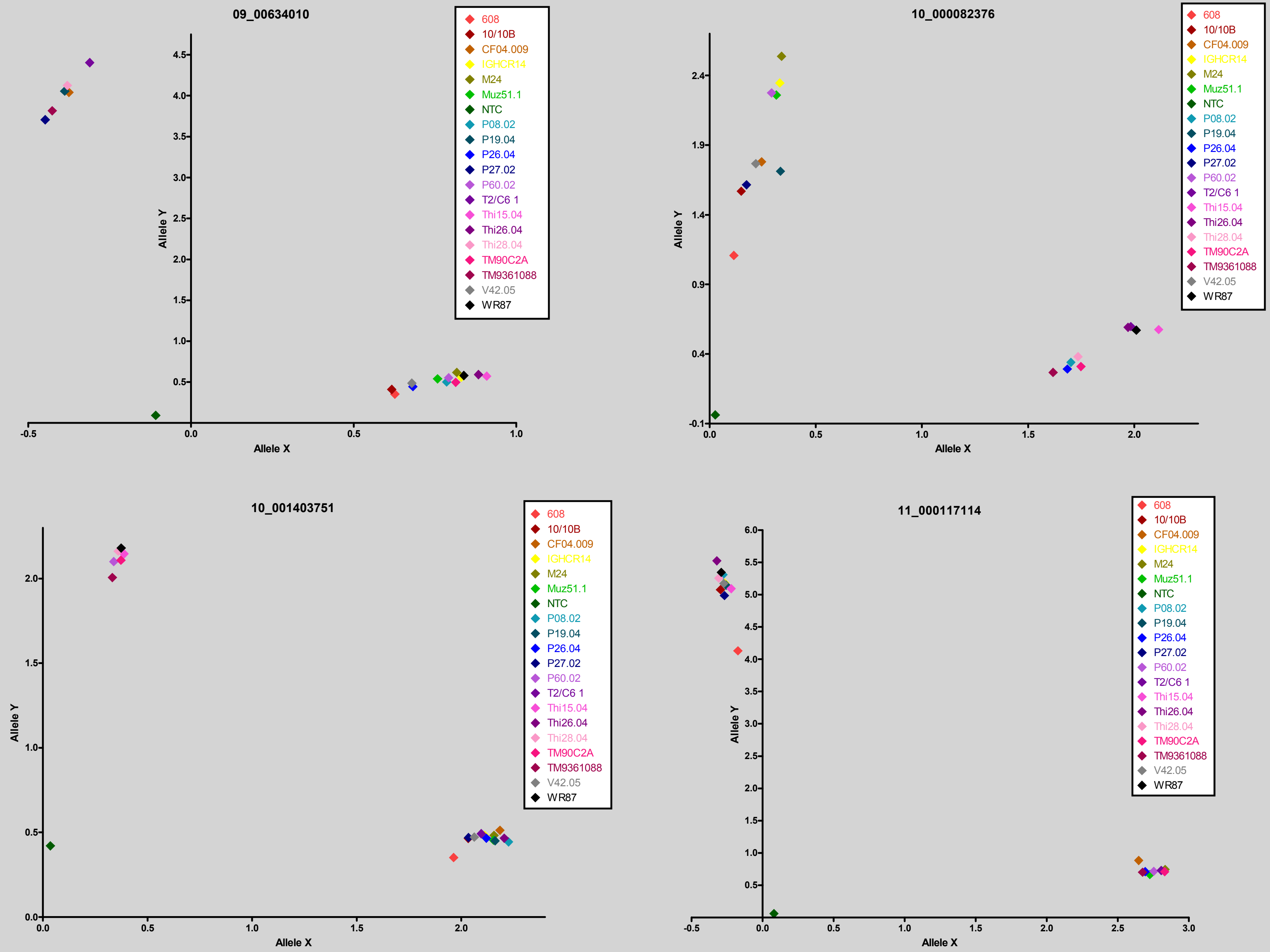

Relative Intensities and Allelic Discrimination: Assay 21 - 24

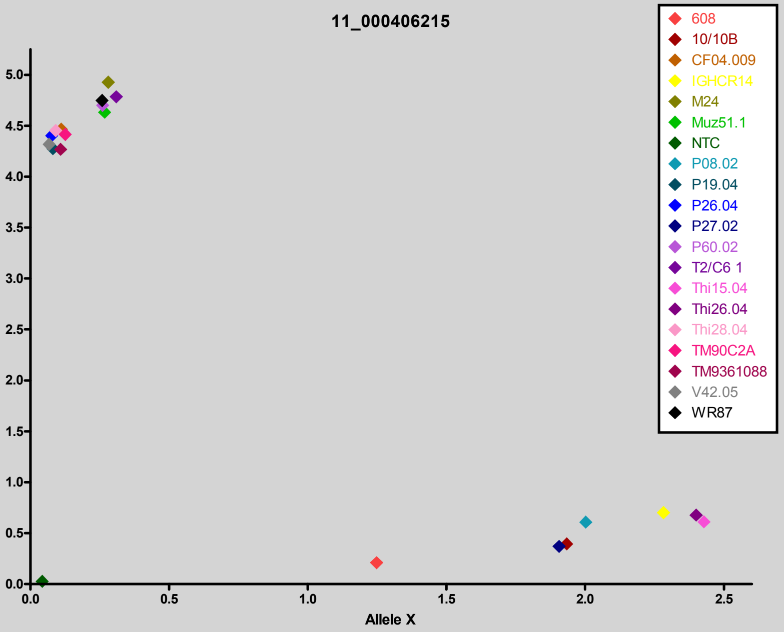

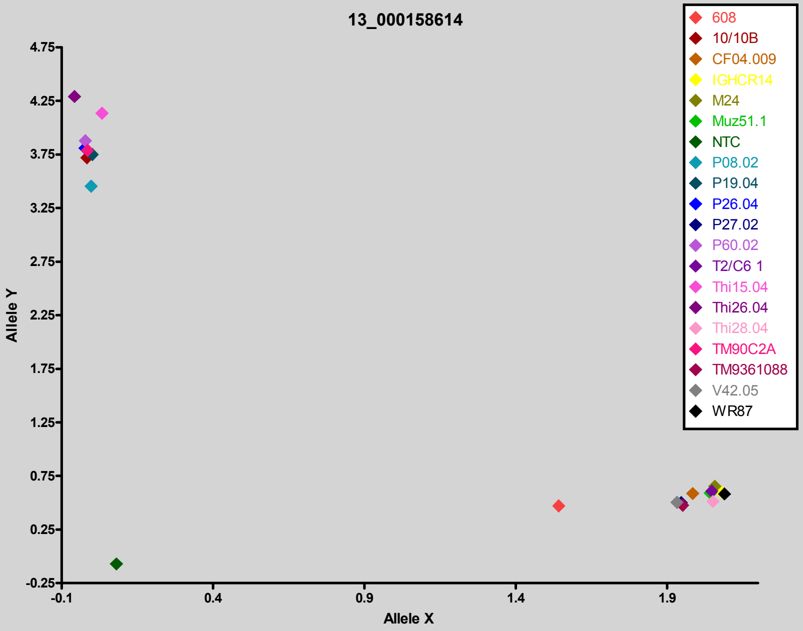

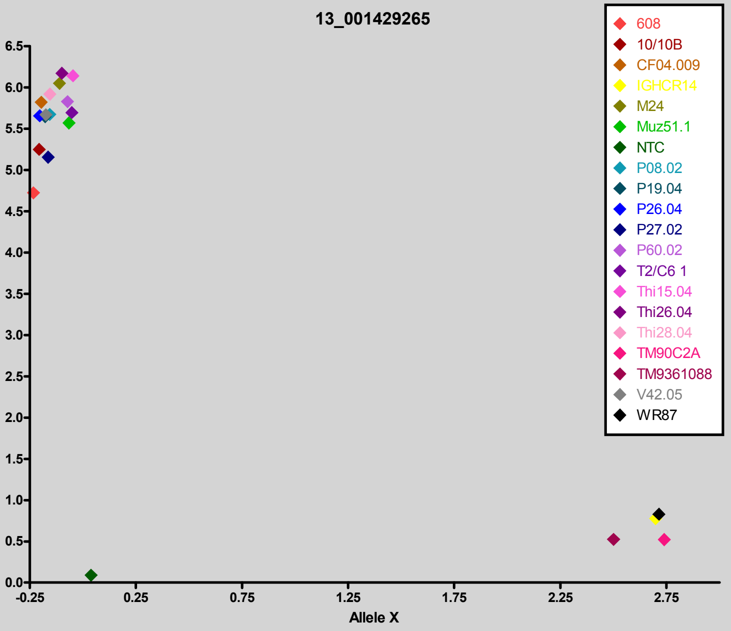

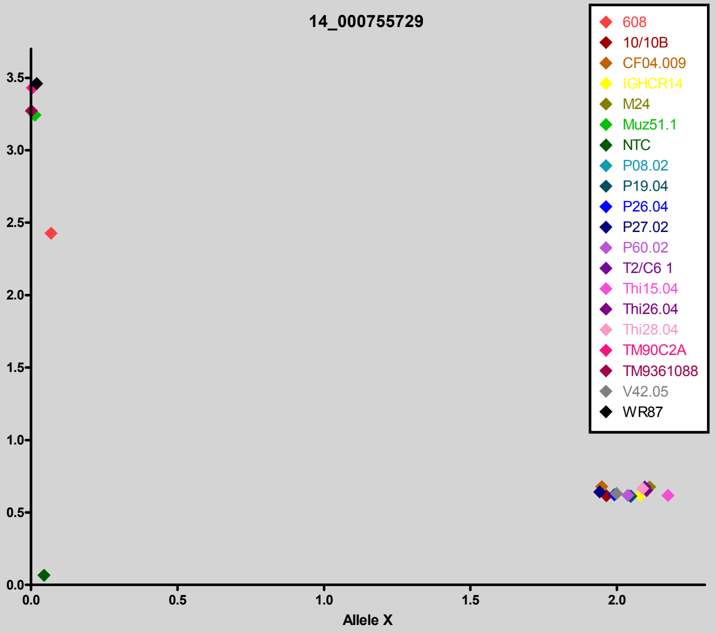

Supplement: Additional file 6 — Allele discrimination data (individual) for all 24 probes on 20 isolates. Allele discrimination assays run for a subset of the parasites (corresponding to a typical running of the assay) are shown, indicating the clear separation between the signal derived from the major and minor allele. The major allele (Allele X) is displayed on the X axis and the minor allele (Allele Y) on the Y axis for 20 independent strains along with a non template control (NTC) containing only water. [file 1475-2875-7-223-S6.pdf]
