# Supplementary material for: A general SNP-based molecular barcode for Plasmodium falciparum identification and tracking
Source: Malar J. 2008 Oct 29;7:223. doi: 10.1186/1475-2875-7-223 (PMC2584654; doi:10.1186/1475-2875-7-223)
Supplement: Additional file 8 — Assay performance. The performance of each assay was evaluated by assessing the lowest ratio (20:1 in dark green; 10:1 in light green; and 5:1 in red) where the X or Y allele could be detected in a mixture containing both the major and minor allele, with the reported performance for the assay being the highest ratio for detecting either the X or Y allele in a mixture. [file 1475-2875-7-223-S8.pdf]

	Mixture Detection X allele	*Mixture Detection Y allele*	*Reported Performance*
Pf_01_000130573	5:1	20:1	5:1
Pf_01_000539044	10:1	20:1	10:1
Pf_02_000842803	20:1	5:1	5:1
Pf_04_000282592	10:1	5:1	5:1
Pf_05_000931601	10:1	10:1	10:1
Pf_06_000145472	10:1	10:1	10:1
Pf_06_000937750	20:1	10:1	10:1
Pf_07_000277104	20:1	20:1	20:1
Pf_07_000490877	20:1	20:1	20:1
Pf_07_000545046	20:1	20:1	20:1
Pf_07_000657939	20:1	5:1	5:1
Pf_07_000671839	10:1	20:1	10:1
Pf_07_000683772	10:1	10:1	10:1
Pf_07_000792356	10:1	20:1	10:1
Pf_07_001415182	10:1	20:1	10:1
Pf_08_000613716	10:1	10:1	10:1
Pf_09_000634010	10:1	20:1	10:1
Pf_10_000082376	20:1	10:1	10:1
Pf_10_001403751	5:1	20:1	20:1
Pf_11_000117114	10:1	20:1	10:1
Pf_11_000406215	20:1	20:1	20:1
Pf_13_000158614	10:1	20:1	10:1
Pf_13_001429265	10:1	20:1	10:1
Pf_14_000755729	10:1	20:1	10:1
